# Trello

**DOI:** 10.29173/jchla29545

**Published:** 2021-04-02

**Authors:** Vanja Stojanovic

**Affiliations:** Co-op Librarian, Saskatchewan Health Authority Library, Regina, Saskatchewan, Canada

## Abstract

Trello is a project management solution that supports real-time collaboration and communication to accomplish project tasks and goals. Ideal for small-to-medium sized libraries, Trello’s intuitive user interface and robust integrations provide teams the flexibility to customize their workflows to meet their unique needs while delivering value to their user communities. Available with a free or paid subscription, this tool enhances teamwork and provides control over simple and complex projects.

**Product:** Trello

**URL:** https://trello.com/

**Cost**

Free and tiered pricing available

## Purpose and Intended Audience

Trello is a web-based and mobile-friendly project management tool intended to enhance collaboration and productivity. Its purpose is to support the organization, visualization, and tracking of project tasks for individuals and teams alike. Its simple design and customizable features have been a boon for a variety of knowledge workers, not least of which are librarians and information specialists coordinating information literacy programs, literature search requests, electronic resources, and other services [1, 2].

## Product Description

**Fig. 1:**
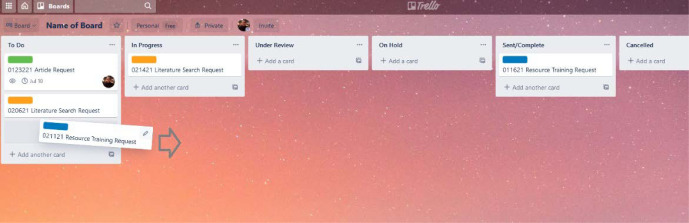
A Trello ‘board’ showing a ‘card’ being dragged and dropped into the “In Progress” list.

As a *Kanban*-style project management tool, Trello’s design allows users to create ‘boards’ upon which they may delineate columns (referred to as ‘lists’) for each step of a given project (e.g., to do, in-progress, under review, etc.), usually in a sequential manner from left to right. Within these columns, ‘cards’ may be created to capture specific tasks and any associated information such as the task description, its due date, and the team member assigned to the task. These cards may then be moved by dragging and dropping them into other lists when the task is completed, or its status changed to the next step in the process (Figure 1).

**Fig. 2:**
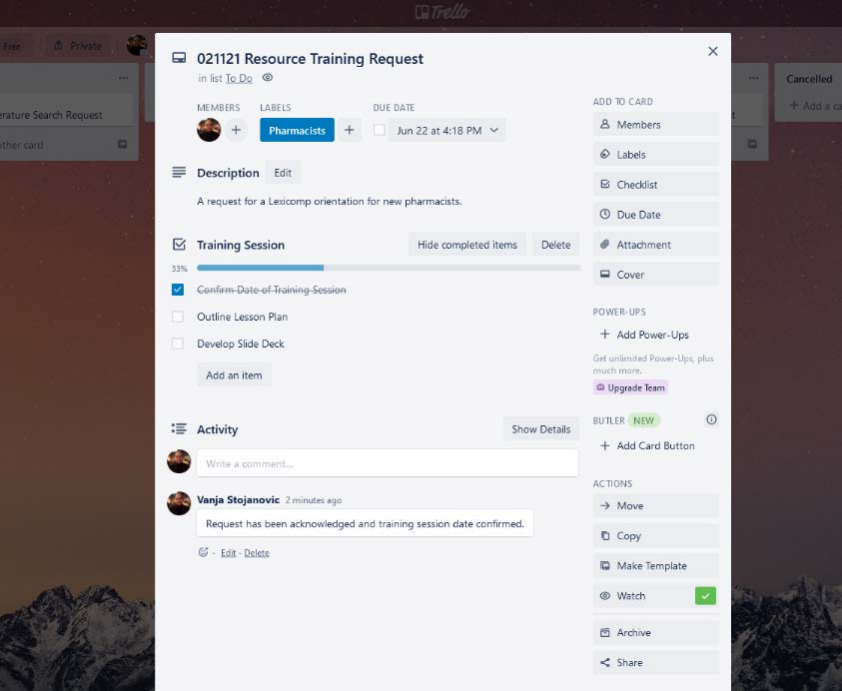
The “back” of a single Trello ‘card’ showing additional details.

Much like using a whiteboard and sticky notes, Trello enhances collaboration through an interactive interface, providing teams with a bird’s-eye view of a project to help identify progress, workload imbalances, deadline expectations, and task bottlenecks. In addition, when used as a regular element of teamwork, Trello can ensure that unexpected or new tasks are captured immediately, mitigating the possibility of tasks “falling through the cracks.” Communication is made easy through a comment function on the ‘back’ of each card where users may add noteworthy information or updates to keep the team informed and up to date (Figure 2). Finally, while cards are typically designated as tasks, they may be adapted to represent a discrete deliverable such as a single article request, or a specific instructional video as it moves through the stages of planning and production.

## Special Features

A variety of customizable features make Trello adaptive and easy to use. For example, setting up a project board includes options to invite team members and to set the board’s visibility to private, just the team, the wider organization, or the public. In addition, users may add labels to cards, create checklists, and even attach documents. One of the most useful features is the template function which allows users to consistently generate new cards that include pre-selected fields relevant to the project (e.g., specific checklists, description fields, etc.). As projects grow, team members may choose to “watch” cards to stay informed about their progress with the option to receive an email notification when changes are made, or the card is moved. Trello also offers a wide variety of “Power-Ups” which are additional features that can be added to individual boards. “Power-Ups” fall under categories such as analytics and reporting, file management, utilities, communication and collaboration, developer tools, marketing and social media, and many others. Finally, Trello’s “Butler” feature is an automation function that enables users to streamline workflows by setting up buttons, rules, and scheduled commands based on user interaction, helping to minimize clicks toward accomplishing tasks [3].

## Compatibility & Integration

Trello is compatible across various web browsers such as Chrome, Safari, Firefox, and Microsoft Edge. In addition, it is available as a mobile app for both iOS (version 13 or higher) and Android (version 6.0 or higher) devices. Although a popular web-based tool, Trello is also available as a desktop app for macOS (version 10.9 or higher) and Windows (version 10) computers. Adding to its customizability, Trello allows for the integration of other tools such as Slack, MailChimp, Google Drive, Microsoft Teams, GitHub, Twitter, and many others.

## Usability

Trello is user-friendly and intuitive in its design and interactive functionality. The user experience is visually engaging, yet simple in its overall design. Users may select from a menu of images or colours to customize the board background and may also add a profile photo to their account. In addition, a search bar enables easy discovery of information across cards and boards. There is a low learning curve in its basic functionality, however any “Power-Up” additions may require users to spend some time in determining the best ways to integrate them into specific workflows.

## Strengths

- Enhances project management workflows.
- Visualizes project steps and statuses.
- Presents an easy-to-use and customizable interface.
- Enables real-time collaboration, communication, and sharing.
- Supports compatibility and integration across major platforms and tools.
- Accessible in over 25 different languages.

## Weaknesses

- Limit of 10 boards per team with free account.
- Limit of 10MB per file attachment with free account.
- Data exporting functions only available for Business Class and Enterprise subscriptions.
- Limit of one (1) “Power-Up” with free account.
- A high volume of cards may require users to scroll through lists, resulting in some cards being out-of-sight.

## Comparison with Similar Products

Similar tools include Monday.com, Kanbanize, Asana, Wrike, as well as Todoist which includes Kanban functionality. Overall, each of the above tools is on par with the basic functionality offered by Trello, though Trello is a crowd favorite for its diverse integration options.

## Cost

Trello is a subscription-based tool available in three pricing tiers: 1) Free, 2) Business Class ($9.99 USD per user, per month), and 3) Enterprise (as low as $17.50 USD per user, per month depending on the size of the organization) [4]. Successive tiers provide enhanced “Power-Ups,” additional integration options, advanced automation, increased security features, and data management capabilities.

## Conclusion

In a small-to-medium health sciences library, Trello is an easy and collaborative solution to managing projects of any size. Whether tracking the progress of working groups or managing electronic resource licences, Trello has the potential to boost workflow efficiencies and communication. Ultimately, this tool adds value by easing project management tasks, allowing librarians and information professionals to focus on delivering exceptional services to their users.
